# Sun Safety at Work Canada: a multiple case-study protocol to develop sun safety and heat protection programs and policies for outdoor workers

**DOI:** 10.1186/s13012-015-0277-2

**Published:** 2015-07-10

**Authors:** Desre M. Kramer, Thomas Tenkate, Peter Strahlendorf, Rivka Kushner, Audrey Gardner, D. Linn Holness

**Affiliations:** 1 Occupational Cancer Research Centre, Cancer Care Ontario, 3rd floor, 525 University Avenue, Toronto, ON M5G 1X3 Canada; 2 School of Occupational and Public and Health, Ryerson University, 350 Victoria Street, Toronto, ON M5B 2K3 Canada; 3 St Michael’s Hospital, 30 Bond St., Toronto, ON M5B 1W8 Canada

**Keywords:** Solar UV, Knowledge translation, Workplace-based research, Implementation, Case study design, Occupational health and safety

## Abstract

**Background:**

CAREX Canada has identified solar ultraviolet radiation (UV) as the second most prominent carcinogenic exposure in Canada, and over 75 % of Canadian outdoor workers fall within the highest exposure category. Heat stress also presents an important public health issue, particularly for outdoor workers. The most serious form of heat stress is heat stroke, which can cause irreversible damage to the heart, lungs, kidneys, and liver. Although the need for sun and heat protection has been identified, there is no Canada-wide heat and sun safety program for outdoor workers. Further, no prevention programs have addressed both skin cancer prevention and heat stress in an integrated approach. The aim of this partnered study is to evaluate whether a multi-implementation, multi-evaluation approach can help develop sustainable workplace-specific programs, policies, and procedures to increase the use of UV safety and heat protection.

**Methods/design:**

This 2-year study is a theory-driven, multi-site, non-randomized study design with a cross-case analysis of 13 workplaces across four provinces in Canada. The first phase of the study includes the development of workplace-specific programs with the support of the intensive engagement of knowledge brokers. There will be a three-points-in-time evaluation with process and impact components involving the occupational health and safety (OHS) director, management, and workers with the goal of measuring changes in workplace policies, procedures, and practices. It will use mixed methods involving semi-structured key informant interviews, focus groups, surveys, site observations, and UV dosimetry assessment. Using the findings from phase I, in phase 2, a web-based, interactive, intervention planning tool for workplaces will be developed, as will the intensive engagement of intermediaries such as industry decision-makers to link to policymakers about the importance of heat and sun safety for outdoor workers.

**Discussion:**

Solar UV and heat are both health and safety hazards. Using an occupational health and safety risk assessment and control framework, Sun Safety at Work Canada will support workplaces to assess their exposure risks, implement control strategies that build on their existing programs, and embed the controls into their existing occupational health and safety system.

## Background

### Skin cancer and heat stress

The global incidence of skin cancers (both cutaneous malignant melanoma (CMM) and nonmelanoma skin cancers (NMSCs)) has continued to rise over the last decade with up to 3 million cases of NMSCs and 132,000 cases of CMMs occurring each year [1]. This means that one in every three cancers diagnosed is a skin cancer [1]. For Canada, the incidence rate of skin cancers continues to increase. NMSCs are the most commonly diagnosed cancer, with an estimated 76,100 cases and 320 deaths in 2014. In addition, it is estimated that there will be 6,500 new cases of and 1,060 deaths from melanoma in Canada in 2014 [2]. From an economic perspective, the direct and indirect costs of skin cancer for Canada have been estimated to be over $531 million in 2004, with this number projected to rise to over US$921 million by 2031 [3]. Heat stress also presents an important public health issue, particularly for outdoor workers [4]. The most serious form of heat stress is heat stroke, which can cause irreversible damage to the heart, lungs, kidneys, and liver; it has been found to be associated with increased risk of cardiovascular disease, ischemic heart disease, and chronic liver and renal failure.

The most important risk factor for developing skin cancer is exposure to ultraviolet (UV) radiation [5, 6], with occupational solar exposure contributing greatly to overall lifetime UV dose and resulting in an excess risk of skin cancer [7]. The European Community has also identified occupational exposure to UV radiation as one of the most important physical risks in the work environment [8]. Therefore, when the impacts of heat-related conditions are considered in combination with skin cancer, the importance of effective occupational sun safety becomes apparent.

Typical outdoor worker exposures are between 30 and 50 % of the ambient UV levels [9], but these can rise to over 100 % of the ambient UV level in occupations that work in highly reflective environments [10]. No studies on personal UV exposure of outdoor workers have been undertaken in Canada [11]. Outdoor workers tend to spend a significant proportion of their workday in the sun, e.g., in Canada, 67 % of outdoor workers spend two or more working hours in the sun daily [12]. However, workers in the construction, agriculture, forestry, and fishing sectors report even higher daily exposure [13]. In Canada, there are between 1.5 [11] and 5.4 million outdoor workers [12], with CAREX Canada identifying solar UV as the second most prominent carcinogenic exposure in Canada following shift work. However, unlike shift work, solar UV is a known human carcinogen with over 75 % of Canadian outdoor workers falling within the highest exposure category [14].

### Occupational sun safety measures

The World Health Organization recommends that sun protection be provided once the UV index exceeds 3 [15]. For Canada, this means UV protection should be used between April and August in many locations (e.g., in Edmonton), with this extending from March through October in some locations (e.g., in Toronto) [16]. For outdoor workers, general protection measures include the following: engineering controls (for example, the provision of shade, window tinting); administrative controls (educational programs, recognition of individual susceptibilities, sun avoidance strategies, and scheduling of work hours outside of peak UV times); and personal protective equipment (PPE) (wide-brimmed hats, long-sleeved shirts and long pants, use of sunscreen, and eye protection) [17]. These measures should be part of a broader risk management approach that also includes implementing workplace policies and a risk management process [18].

Despite the wide-spread availability of educational materials and sun safety resources (for example, the Canadian Dermatology Association’s Sun Safety for Outdoor Workers Manual or *Be Sunsible*www.besunsible.ca and other resources [19–21]) and the easy availability of a wide range of protection measures, numerous studies have shown that implementing effective sun protection programs for outdoor workers continues to be highly challenging. Few workers are considered to be adequately protected (in some cases 10 % or less) [10, 22–28] with the face and lower arms being the least protected sites [24, 29] and the use of sun screen and wide brimmed hats particularly low [23, 25–27, 30–32]. As such, improving sun protection among outdoors workers is a strategic objective under the Canadian Strategy for Cancer Control [33] and is a key recommendation from the Second National Sun Survey [34].

Control measures for heat stress are well defined and include acclimatization, engineering controls, administrative and work practice controls, and protective clothing [35]. Many of these measures are routinely implemented; however, outdoor workers often have low levels of knowledge regarding appropriate work practices, such as the amount of time required to appropriately acclimatize to hot conditions and the amount of water they should consume [36, 37]. There is also the need to improve workers’ understanding on the benefits of long clothing, including that it does not increase body temperature while providing UV protection [38].

### Sun safety interventions/programs

For many years, there have been community-wide sun safety promotion programs that have focused on skin cancer prevention. A recently completed dynamic simulation modeling exercise to assess the range of sun safety interventions available in four European countries concluded that future interventions should focus on protecting outdoor workers [39]. In the USA and Canada, comprehensive reviews on the effectiveness of sun safety/skin cancer prevention programs have been undertaken [40, 41]. In the USA, the Community Preventive Services Task Force currently recommends multi-component community-wide interventions for skin cancer prevention along with education and policy approaches in outdoor recreation settings [42]. This task force recently updated their advice and, based on the strength of evidence, they now recommend educational interventions that promote sun protective behaviors in outdoor occupational settings to prevent skin cancer. However, evidence gaps still exist regarding the effectiveness of interventions with worksite policy components, such as those that target non-White outdoor workers, and interventions that evaluate health outcomes [43]. A review conducted on behalf of the Canadian Partnership Against Cancer (CPAC) found that there are a range of strategies that are effective for skin cancer prevention, but that success criteria are not currently entirely understood [40]. The National Cancer Institute in the USA maintains the Research Tested Intervention Programs (RTIPs) database that contains details of programs and relevant materials. For sun safety, 17 programs are listed, however, of these, only two relate to outdoor worker interventions (http://rtips.cancer.gov/rtips/index.do).

Five systematic reviews have been conducted in recent years on sun safety interventions for outdoor workers [41, 44–47]. These have concluded that up until recently, there was insufficient evidence to determine the effectiveness of interventions in this setting due to the limited evidence base. However, there is now growing evidence for the effectiveness of sun safety interventions for outdoor workers, while considerable room for improvement remains in occupational sun protection indicating that more research is needed on this topic. In particular, for greatest possible impact, the evidence indicates that a comprehensive workplace strategy that includes multiple interventions aimed at both workers and employers is needed [44]. In addition, there is increasing evidence for the influence of workplace culture on the effectiveness of occupational health and safety interventions [48], and particularly for outdoor sun protection interventions [49]. Six randomized control trials involving outdoor worker sun safety interventions have been identified [22, 50–54]. Two of these studies [52, 53] are included in the RTIPs database. The four most comprehensive intervention projects are SUNWISE (with Southern Californian US Postal Service letter carriers conducted between 2001 and 2004) [53, 55, 56], Go Sun Smart (with employees working at ski areas in North America between 2004 and 2007) [52, 57–61], Pool Cool (with children and lifeguards at swimming pools across the US between 1999 and 2005) [50, 62–66], and the recent QUT Outdoor Workers Project (with small-to-medium rural workplaces in Queensland, Australia) [67, 68].

In summary, although the need for sun and heat protection has been identified, there is a scarcity of projects that have systematically focused on outdoor workers, have addressed both skin cancer prevention and heat stress in an integrated approach, or have specifically investigated occupational sun protection through the lens of integrated knowledge transfer bringing together the expertise of researchers, practitioners, and policymakers.

This paper describes the study protocol for a project funded by the CPAC. CPAC is an independent organization funded by the Canadian federal government that has the mandate to implement the Canadian strategy for cancer control. It funds research projects that are federal and collaborative (among other initiatives). This study was funded under its research program called Coalitions Linking Action and Science for Prevention (CLASP), which is now in its second phase of funding. This project was one of eight that was funded to address issues such as obesity, tobacco use, screening for cancer and chronic disease, and the unique health needs of First Nation communities.

This paper describes the rational and methodology for a workplace-based implementation study for integrated sun and heat safety programs with multiple workplaces. Using an occupational health and safety risk assessment and control framework, the project, called Sun Safety at Work Canada, supports workplaces to assess their exposure risks, implement control strategies that build on their existing programs, and embed the controls into their existing Occupational Health and Safety system. The study also includes a roll-out to raise awareness across workplaces of the danger to outdoor workers of UV exposure through an interactive website and intensive engagement with workplace and policy decision-makers.

## Methods/design

### Research study aims

The aim of the study is to create a nationally applicable sun safety program. The short-term outcomes expected from the project relate to improved employee and employer awareness of the importance of sun safety. This is within the context of improved employer support for sun and heat safety that is demonstrated through the development and implementation of sun safety policies and programs and the commitment of resources to support these policies/programs. The medium-term desired outcomes relate to providing conditions that will enable sun safety programs to be implemented in a sustainable manner. This includes influencing policy and practice to support and enforce workplace sun safety. The long-term desired outcome is the decrease of skin cancer risk and heat-related illness risk for outdoor workers.

#### Two-phased intervention design

The project takes an integrated knowledge translation (IKT) approach that brings together researchers, policy advocates, and practitioners throughout the research process. The partners are Ryerson University’s School of Occupational and Public Health, the Occupational Cancer Research Centre, Sun Safe Nova Scotia, the Occupational Health Nurses Association Nova Scotia, CAREX Canada, Canadian Cancer Society Nova Scotia, Centre for Research Expertise in Occupational Disease, and Alberta Health Services. The study partners have been involved and engaged in the initial conception of the study, the recruitment of workplaces and Sun Safety Advisor knowledge brokers, the creation of the sun safety program suite of resources for workplaces, determining the data that will be collected at three points in time during the study, and the dissemination phases of the study. The partners are based in the four Canadian provinces (Ontario, Nova Scotia, New Brunswick, and British Columbia) in which the interventions will take place and local partners will take the lead in supervising the local interventions.

The research team is interdisciplinary in nature, with researchers from epidemiology, occupational health and safety, occupational medicine, law, sociology, knowledge translation, and adult education. The study uses a mixed methods approach, with the qualitative data analysis being used to help inform and gain a better understanding of the quantitative findings [69]. The project has two complementary phases of activities, in which phase 2 builds on the outcomes of phase 1. Phase 1 is characterized by the intensive engagements with 13 workplaces across Canada and a three-phased evaluation: before the implementation, after the first summer, and 18 months after the project starts (including a second summer). Phase 2 includes an extensive outreach to industry and not-for-profit decision-makers and organizations who are interested in sun protection and policy-level discussions with industry decision-makers, and the creation of a website with tailored resources for workplaces. The process and impact evaluations will be driven by conceptual frameworks to ensure that the phases are theoretically linked and to help us to understand the knowledge translation needs and requirements in rolling out a sun and heat protection programs and policies in workplaces across Canada.

#### Guiding frameworks

**Fig. 1:**
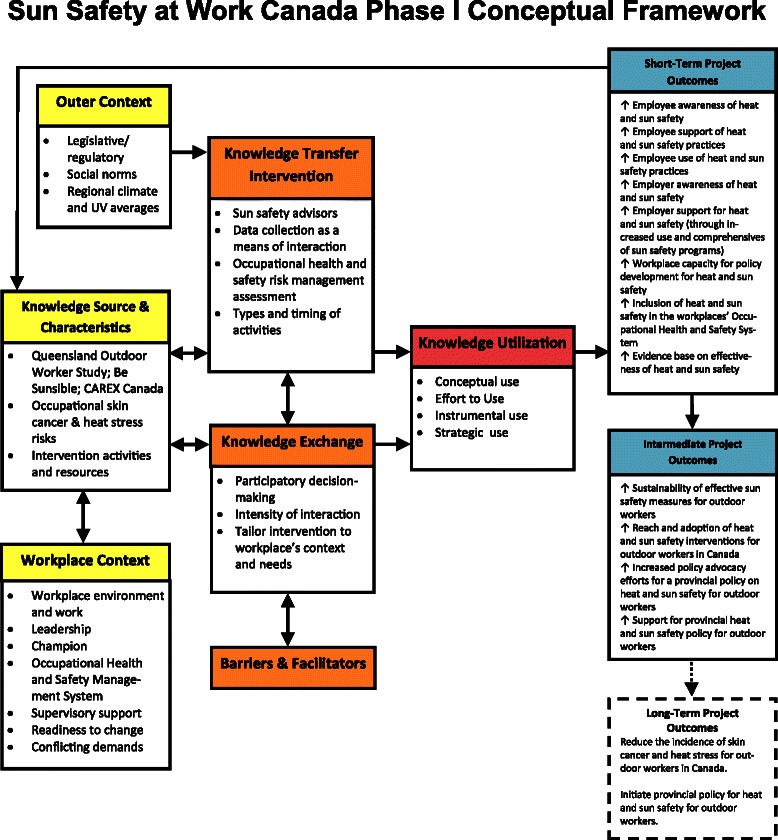
Sun Safety at Work phase I conceptual framework

**Fig. 2:**
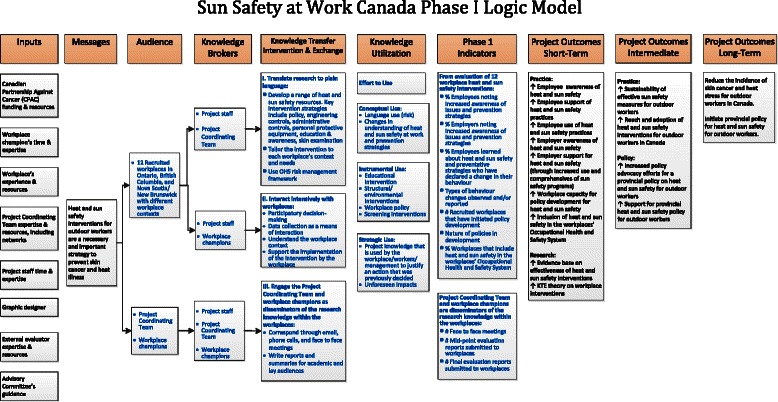
Sun Safety at Work phase I logic model

We have adopted three frameworks to guide our study. Versions of them have been used by different research studies in the field of knowledge translation and implementation science. Phase 1 of the study will be framed by a knowledge transfer, dissemination, and utilization in organizations conceptual framework (see Fig. 1). The framework and its matching logic model (see Fig. 2) will drive the evaluation of the effectiveness of the individual workplace implementations. This framework was developed specifically for workplace-based occupational health and safety initiatives [70, 71] by one of this project’s team members (DK) and has recently been used by Allen et al. [72]. The model’s five variables focus on the outer context, the knowledge source and characteristics, the workplace context, the facilitation of the knowledge transfer, indicators of knowledge exchange, and stages of knowledge utilization. The indicators of the latter will be conceptual, effort to use, procedural, and structural use. The short-, medium-, and long-term outcomes focus on the workplaces’ structural, policy, and educational changes.

**Fig 3:**
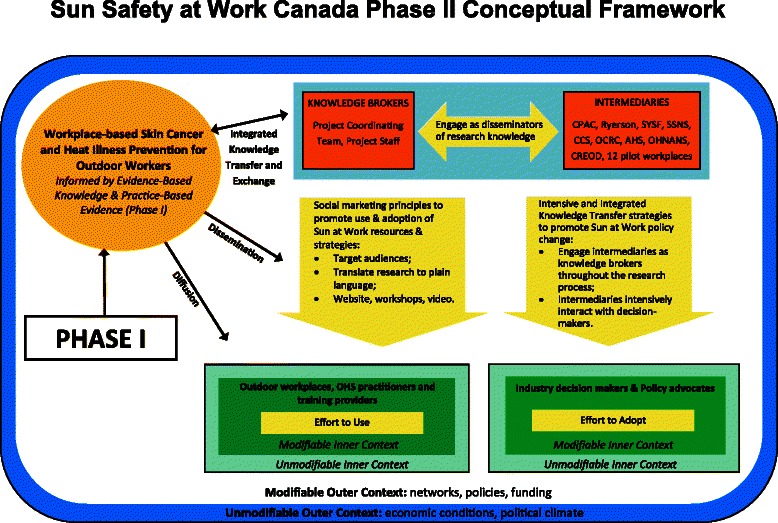
Sun Safety at Work phase II conceptual framework

**Fig. 4:**
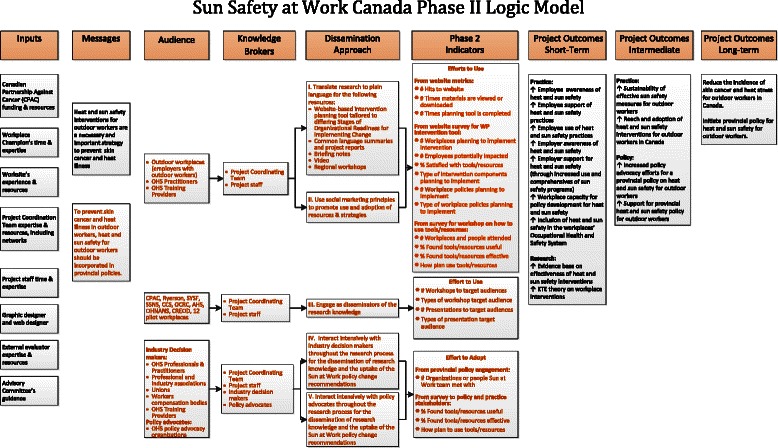
Sun Safety at Work phase II logic model

Phase 2 of the study will be framed by a dissemination and knowledge transfer framework (see Fig. 3) and its matching logic model (see Fig. 4). The evidence-based knowledge that drives this dissemination model will be informed by the findings from phase 1. This framework is an adaptation of Harris et al.’s [73] and emphasizes the relationship between researchers, disseminating organizations, and user organizations for successful knowledge exchange [73]. The user organizations for this project will include workplaces, industry and not-for-profit decision-makers and organizations, and policy advocates. The setting of the knowledge dissemination will include modifiable variables such as relationships and networks, workplace policies, and funding allocations, and will take into account unmodifiable factors such as the economic and political climate. This framework incorporates principles of social marketing in the dissemination approach and intensive and integrated knowledge transfer strategies, which align closely with the objectives of phase 2.

**Fig. 5:**
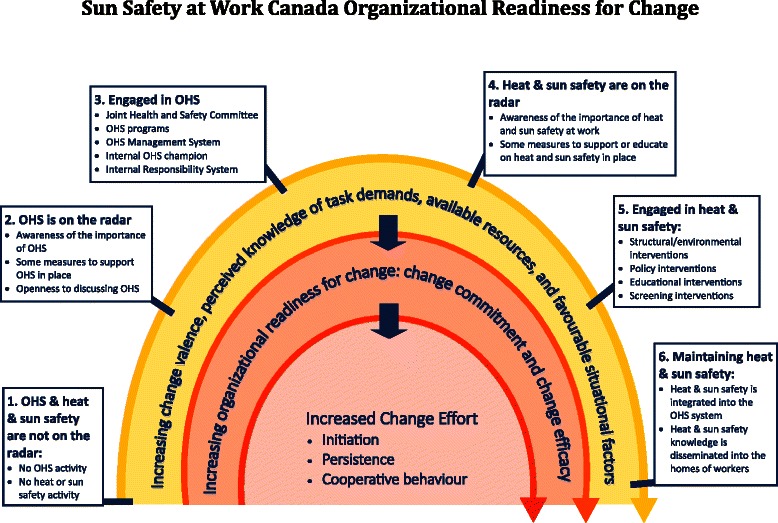
Sun Safety at Work organizational readiness for change model

In the experience of the researchers, a determination of readiness to change is essential particularly for phase 1 of the study since it is a major driver of the success of any workplace-based intervention [74]. Hence, an organizational readiness for change “Sun” model (see Fig. 5) was used especially for the recruitment of workplaces. It was used as a communication tool by the study partners to help explain the project to potential workplaces. The model is based on the transtheoretical model [75] and an adaptation of Shea et al.’s ”Determinants and outcomes of organizational readiness for change” model [76]. The model takes into account workplaces’ perception that change (on sun and heat protection) is needed, important, worthwhile, or beneficial, and their self-appraisal that they have the needed resources to make the changes [77]. We conceptualize workplaces to be at one of five possible stages of readiness to implement sun safety interventions for outdoor workers: (1) pre-contemplation: occupational health and safety (OHS) is not on the organization’s radar; (2) contemplation: OHS is on the organizational members’ radar; (3) preparation: the organization is engaged in OHS; (4) action: sun safety is on the organization’s radar; and, (5) engaged: the organization is already engaged in sun safety practices. In our workplace recruitment, workplaces have self-identified where they believe they fit on the continuum.

We will also create an evaluation framework in order to compare the 13 case studies. The cases will be compared on industry, size of workplace, readiness to change, the interventions they choose, the barriers and facilitators, and changes the three-points-in-time evaluation. The major outcomes for the evaluation are the changes in policy, procedures, and practices at the workplace level.

#### Phase 1: workplace-based sun-safety intervention

The aim of phase 1 is to expand upon the success of existing sun-protection programs by leveraging their findings into the development of a nationally-applicable Canadian-based workplace sun safety program. In this first phase of the study, we will recruit 13 workplaces across four Canadian provinces (i.e., British Columbia, Ontario, Nova Scotia, and New Brunswick) and conduct intensive implementations with the help of Sun Safety Advisors in the role of knowledge brokers. We will work with these workplaces over a period of approximately 18 months.

#### Sampling and recruitment of workplaces

The study is focusing on workers employed with utilities such as electrical distribution companies and those employed with medium-to-large municipalities and provincial wilderness parks. The initial criterion will be whether the organization has (at least) 20–100 employees engaged in outside work. Such outside work will be primarily of a brush-clearing, grounds-keeping, or landscaping nature (i.e., employees undertaking outdoor work on “soft” surfaces). It is anticipated that many utilities will have employees engaged in clearing vegetation from right-of-ways and trimming trees and shrubs near power lines. Municipalities will have employees maintaining municipal properties such as parks and will be engaged in grounds-keeping, lawn-mowing, and landscaping. It is anticipated that some organizations will contract out such brush-clearing or landscaping work and so will not be suitable participants. It is also anticipated that most OHS professionals will have to gain the support or permission of others in the organization in order to participate.

Workplaces were recruited through convenience sampling by using existing contacts of project partners and supporters. A number of the supporters who wrote letters of support for the project are workplaces who are interested in participating in the project; these were contacted through local and regional contacts of the partners on the project. Other potential participants were selected using a database of approximately 10,000 OHS professionals; individuals who occupy the position of OHS “manager,” “coordinator,” “advisor,” “lead”, and so on (there is great diversity of job titles for the individuals who oversee an organization’s OHS efforts). Initial contact was made with the OHS professional employed by a municipality, wilderness park, or utility by senior partners on the study to determine the personal interest of the OHS professional in the aims of the project and to ascertain the numbers and nature of work of the outside workers.

Once this initial contact with the OHS professional had been made, a senior partner on the study attended a group meeting, organized by the OHS professional, of the senior management, operational supervisors, and union representatives. Questions were answered on the purpose of the study, the benefit to the company, the resources that the company will need to dedicate to the project, and how information will be distributed back to management and workers. In general, a letter of voluntary commitment and involvement between the company and the research team completed the recruitment process. However, to ensure that the initial engagement process was respectful to the workplace and to establish a good relationship from the start, the project team worked with each workplace to assist them in gaining the necessary internal approval to participate. As such, whatever form of correspondence for approval was appropriate for each workplace was supported, i.e., we did not use a one-size-fits-all model for gaining workplace sign-on/approval to participate.

**Fig. 6:**
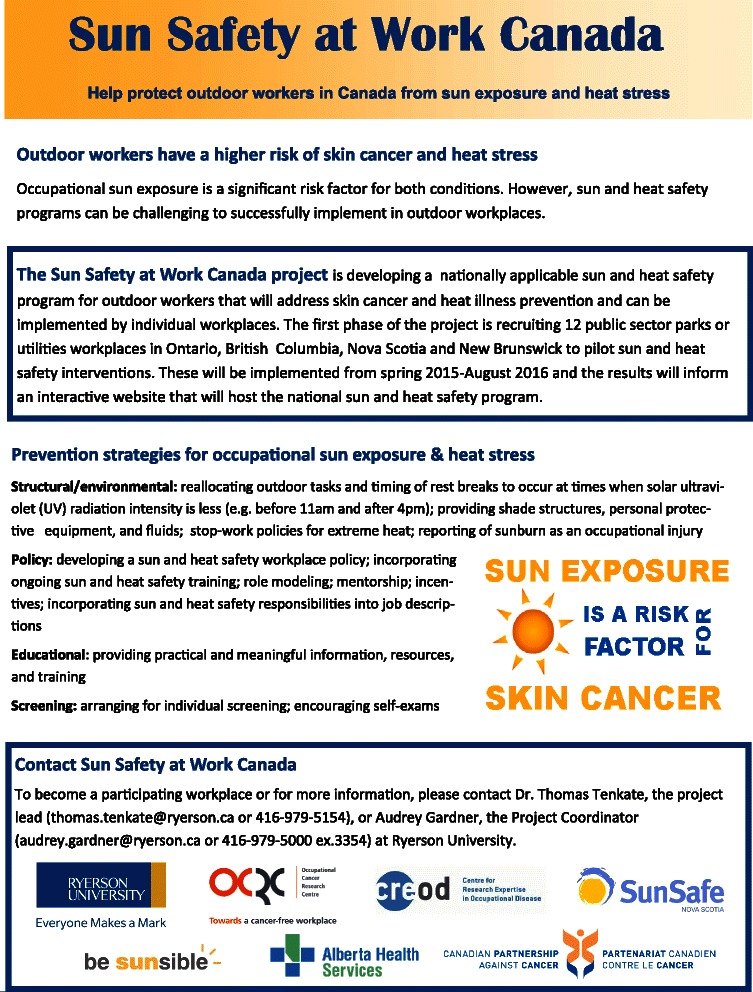
Sun Safety at Work informational flyer for workplaces

**Fig. 7:**
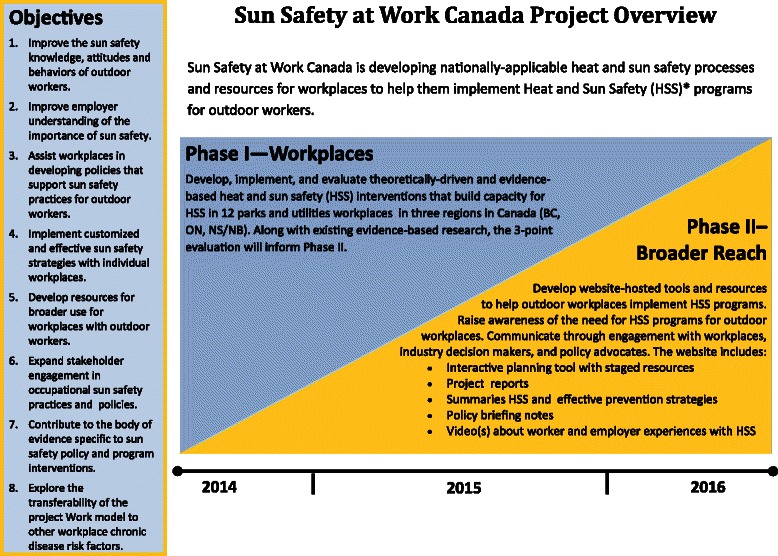
Sun Safety at Work project overview

At the first meeting with the workplaces, they were shown a number of communication pieces on the study, such as an invitation letter, the organizational readiness for change “Sun” model (see Fig. 5), a flyer on the study (see Fig. 6), and an overview diagram of the project (see Fig. 7). These pieces have proved to be very useful to open up the discussion with management and organized labor (if the company is unionized) on their values and beliefs about health and safety in general and sun protection in particular.

#### Recruitment of Sun Safety Advisors

**Fig. 8:**
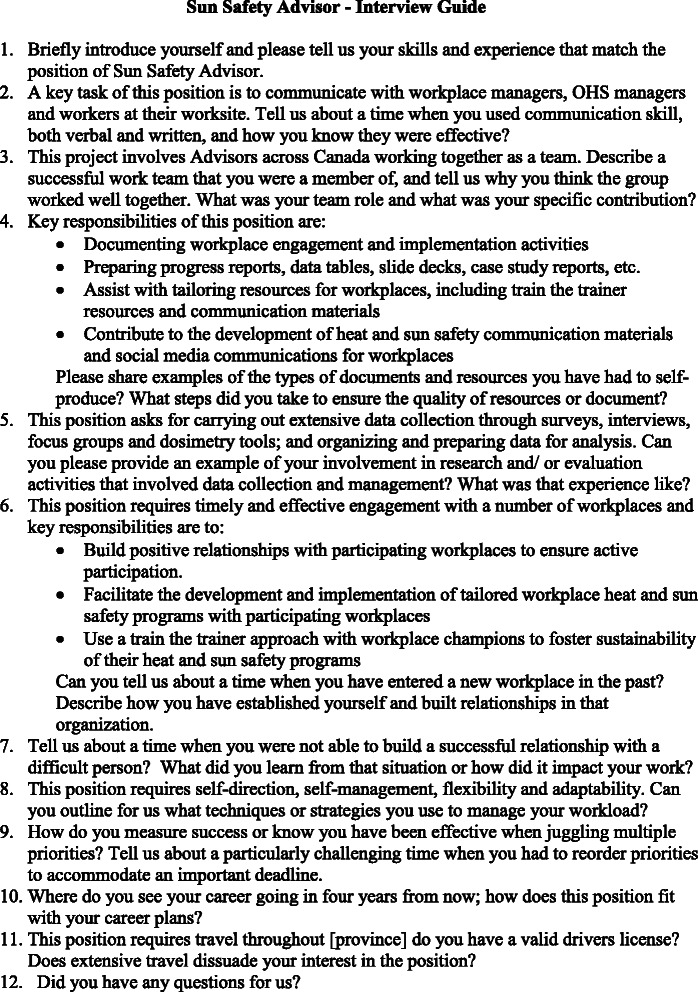
Sun Safety Advisor—interview guide

**Fig. 9:**
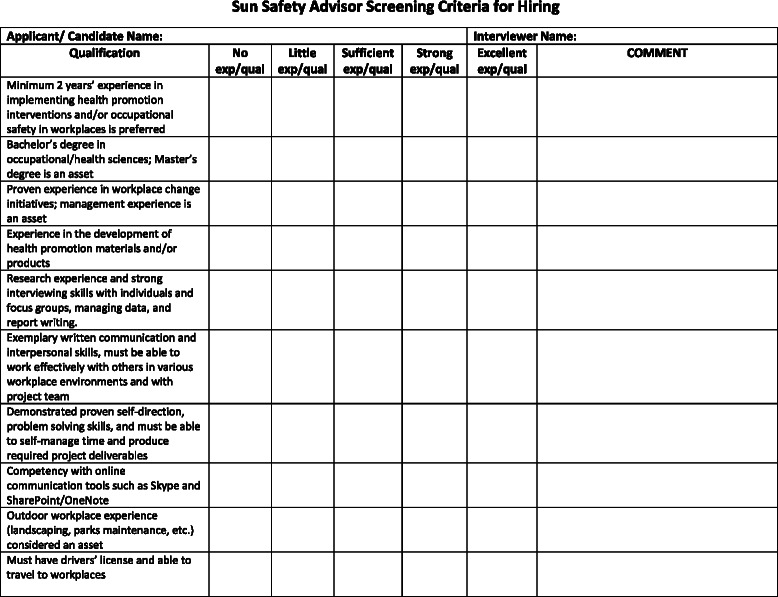
Sun Safety Advisor screening criteria for hiring

**Fig. 10:**
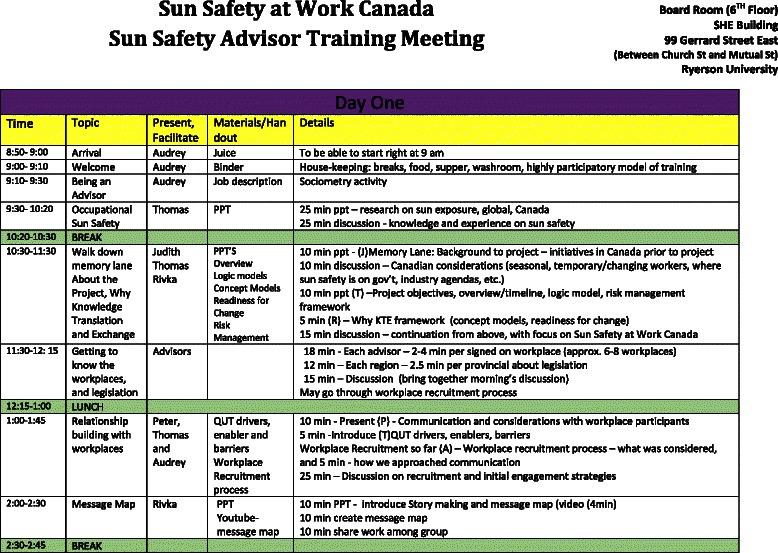
Sun Safety at Work sun safety advisor training meeting

An ongoing positive relationship with the workplaces will be critical to the success of the project. To this end, a project coordinator was hired, and then local project officers (the Sun Safety Advisors) were hired in each province to act as knowledge brokers to build upon this initial recruitment of the workplaces (see Fig. 8 for the interview guide and Fig. 9 for the screening tool that was used during the hiring process). The advisors were selected for their strong background in occupational health and safety, public health, and work with workplaces. They received an initial training in the first quarter of the year to ensure they were all starting with a basic knowledge of the importance of sun and heat protection (see Fig. 10 for the agenda of the training session). The importance of the role of the knowledge broker has been well-explored in the KT literature [78, 79].

The Sun Safety Advisors will interact with one to four workplaces each depending upon geographical convenience. To initiate their relationship with the workplaces, they will meet with their worksites together with the project partner who made the initial contact to reassure the workplace of the level of support offered by the project team. They will engage with the participating worksites (including regular face-to-face contact) with management and workers, and will identify an internal “workplace champion” with whom to work closely. The workplace champion will be the workplace liaison and local project sponsor. The importance of the champion role has also been well explored as a means to ensure sustainability [80]. In previous projects of this type, the workplace occupational health and safety professional has often fulfilled this “workplace champion” role because of their involvement in occupational health and safety projects.

The project officers will collect the evaluation data at base-line before and after the first summer. This includes worker surveys and a face-to-face interview with the health and safety champion and managers. The interview information will focus on assessing the workplace’s organizational readiness for change; the worker surveys will evaluate workers’ awareness and knowledge of heat and sun protective behavior and practices, and when compared with the second evaluation, we will have metrics of change due to the intervention.

#### The Sun Safety at Work implementation

The Sun Safety Advisors will help the companies develop their programs and policies (Sun Safety Action Plans), based upon the findings of the base-line surveys and interviews. The action plan will be tailored to suit each workplace and will be embedded within the companies’ occupational health and safety risk assessment and control framework and risk management systems. It will describe the evaluation process of the project and will specify proposed sun safety activities, the timeline for implementation, who is responsible, and how these activities fit into the legal obligation to create a safe workplace. The action plan will be for 3 years and will extend beyond the project’s funding and direct involvement to ensure sustainability of the initiatives in the workplaces.

The action plan will take into account the financial and organization capacity of the workplace to implement the activities, and depending upon where a workplace is on their sun safety “journey,” will include a phased approach to the implementation of the selected activities. The ability of the workplace to sustain their sun safety program (after the study is over) and their overall stated level of change commitment and sense of efficacy will be a key determinant for the selection of activities and the development of the action plan.

During the first summer, the Sun Safety Advisors will lead the educational initiatives by delivering toolbox talks, showing videos, attending and contributing to joint occupational health and safety committees, giving health and safety meeting presentations regarding sun safety topics, promoting and exhibiting sun safe behavior including the wearing of appropriate PPE, and generally providing current and accurate educational advice to be disseminated to the workforce. They will also advise on how best to implement programs and sun safety change initiatives the employer chooses to undertake and help implement any of the initiatives from a suite of resources proposed by the study.

The suite of resources on different possible interventions has been developed as a group project of the full project coordinating team and the Sun Safety Advisors and the project’s knowledge broker. These materials were selected according to an evidence-based criteria and are based on a comprehensive review of all publically available sun safety resources nationally and internationally and particularly build upon materials developed by the Alberta-based *Be Sunsible* toolkit which identified a five-step process for implementing an effective sun safety program, plus the learnings from an Australian project, the QUT Outdoor Workers Project, which identified an eight-step process for implementing sun safety programs.

The resources include information on policy controls, engineering controls, administrative controls, personal protective equipment, and education and awareness strategies (including personal skin examination strategies). Policy interventions could include the development of a sun safe policy and long-term implementation plan for the workplace. Educational interventions include resources and education about skin cancer and heat stress. Screening interventions include encouraging skin self-examination. One change that has taken place since the original proposal is that the team has decided that organizing individual screening by a dermatologist or medical professional will be too onerous for most workplaces, and it has been dropped as a possible intervention.

To support the workplaces, the plan is to have a project website that will provide a portal for knowledge sharing and access to information and resources. This may include a weekly blog on sun safety topics of interest to the participants and a discussion board for interaction between the workplace champions, employees, and the project team.

#### Phase 1: data collection and analysis

The evaluation will employ a multiple case study approach [81], with the participating workplaces as the units of analysis. The evaluation of the cases will be guided by the conceptual framework for knowledge transfer, dissemination, and utilization for sun safety in outdoor workplaces (Fig. 1) to capture changes in programs, policies, and procedures. Workplace evaluations will occur at three points in time. The baseline assessment will focus on understanding the unique characteristics of each workplace, their occupational health and safety management system, and their current sun safety policy and practices. The second evaluation will be conducted after the first summer (mid-point of the study). The third workplace evaluation will be conducted 18 months after the study’s start date. Finally, a cross-case evaluation matrix will be used to determine generalizable patterns and themes.

**Fig. 11:**
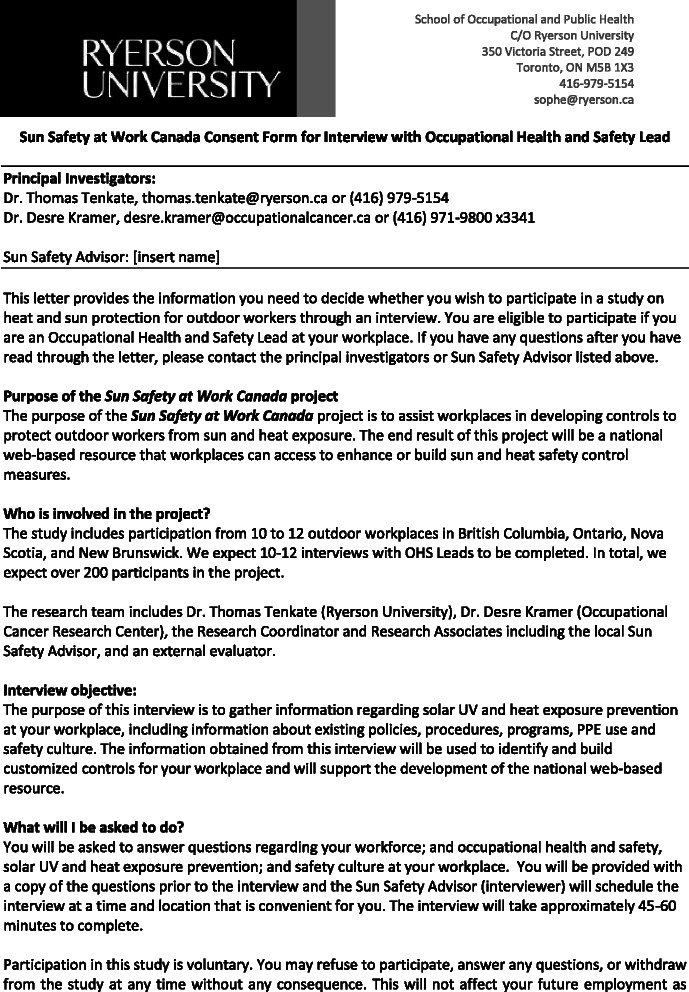
Sun Safety at Work consent form for interview with occupational health and safety lead

Data collection will use mixed methods involving multiple sources of information including semi-structured key informant interviews and focus groups with workers, management, and the occupational health and safety specialist to determine the current occupational health and safety policies and procedure, readiness to change, organizational culture, and barriers/facilitators to sun safety programming (for example, see Fig. 11 information and consent form). The focus groups with employees will explore barriers and facilitators from the perspective of the different workplace parties—workers, supervisors, managers, and occupational health and safety specialists—and a survey will address individual knowledge, attitudes, and behaviors toward sun safety in the workplace. Site observations will record the use of sun safety practices, and a UV dosimetry assessment will measure UV exposure of the employees in the second summer of engagement.

The data collection at baseline and mid-point will be conducted by the Sun Safety Advisors. The data collection at the final time point will occur by an independent researcher. This approach is being undertaken because the involvement of the Sun Safety Advisors in the baseline and mid-point evaluations is considered to be part of their relationship-building and knowledge translation activities with the workplaces.

The conceptual framework (Fig. 1) will guide the interpretation and analysis of the qualitative findings for each of the cases. The qualitative data will be coded and organized by the dimensions of the conceptual framework, including the details of the workplace context, the characteristics of the knowledge transfer intervention, the knowledge exchange, and the workplace’s knowledge utilization, including indicators of conceptual use, effort to use, procedural use, and structural use. Short-term and medium-term outcomes will be noted. Key issues and emergent themes will be identified. The quantitative data (surveys) will be analyzed with SPSS or SAS and will allow for comparisons across the three points in time and allow for cross-case comparisons.

A cross-case analysis of the evaluation data from the 13 workplaces will be conducted in order to determine differences in the interventions, the context, and the barriers and facilitators that led to changes in policies, procedures, and practices on sun and heat protection. The cases will be compared by provincial jurisdiction (12 of the workplaces are in 3 provinces and fall under provincial legislation, and one workplace is federally organized). Although all the provinces have a version of the “General Duty Clause” to protect workers from unsafe work, the different legislative frameworks do lead to different barriers and facilitators. The companies will also be compared by industry (line workers or municipality); size of workplace (small, medium, or large); readiness to change (as determined by the sun conceptual framework); the interventions they chose (for example, the intensity of the interaction with the Sun Safety Advisors, training, and investment in personal protective equipment); the barriers and facilitators that emerged during the implementation; and the changes in programs, policies, and practices that took place over time as noted by the three-points-in-time evaluation. It will facilitate a better understanding of the ways in which the different workplace parties responded to the intervention, and the barriers and facilitators to the adoption of sun and heat protective measures, and enable the team to refine the suite of resources for a broad outreach to companies across Canada.

#### Phase 2: data collection and analysis

The aim of phase 2 is to develop the sun safety program for use by a national audience by developing relationships with policy, practice, and regulatory agencies. This initiative will be based upon the learnings identified from working with the specific workplaces in phase 1. Learnings will relate to the effectiveness of specific sun safety initiatives, with particular reference to the characteristics of the individual workplaces and identified sectors. In this phase of the study, we will use social marketing principles and be guided by a dissemination and knowledge transfer conceptual framework (Fig. 3) to promote the use and adoption of the resources and strategies. The project partners will interact intensively with policy advocates, industry decision-makers, and the workplaces from phase I to engage them as knowledge brokers to disseminate the findings. This interaction has already begun in the three provinces with identifying which organizations, associations, and policymakers should be targeted and kept informed as the study progresses. A knowledge broker, who was hired at the beginning, will lead this initiative.

We will develop a website with materials for workplaces on sun and heat protection. The program will be hosted on an interactive website that will enable workplaces throughout the country to implement effective sun safety policy and practice on their own. Company OHS leads will be able to download targeted material depending upon their current “stage of readiness,” using the organizational readiness for change “Sun” model as a guide (Fig. 5). As such, the website will enable workplaces to adapt the resources to their own needs and characteristics and to their current stage of policy and practice. The resources will help guide workplaces in planning and implementing sun safety practices in a progressive manner.

In addition to these customizable resources, more general resources to support and emphasize the importance of occupational sun safety will be developed and made available on the website. These will include summary statements and resources for use by workers, management, and occupational health and safety practitioners, policy briefing notes for regulators on particular outcomes of the research (from phase I), a series of short videos of worker experiences with skin cancer and heat stress, and videos that record workplaces’ experiences implementing sun safety programs.

As the key outcome of the project is to influence policy and practice, a knowledge translation dissemination strategy for broad reach (i.e., to all Canadian workplaces and workers) will be developed progressively while the project is being implemented and based upon the phase 2 logic model (see Fig. 4). This strategy will allow for the immediate and sustainable exchange and translation of the learnings of the project for a broader workplace audience.

The tools and resources developed for broad reach will be disseminated by the project’s partners and their network of industry decision-makers in a deliberate and coordinated effort to influence policy and practice. This will include using the project’s database of over 10,000 email addresses of OHS practitioners from across Canada, from multiple sectors, as a way of making direct contact to advise them of the tools/resources available, posting information about the tools/resources through social media, hosting workshops in each of the project locations, and having a dedicated website that contains the tools and resources developed for phase 2. A series of regional workshops on the project findings and how to use the developed resources will also be hosted for safety professionals and others from industry and government.

The use and “traffic flow” through the dedicated website will be monitored to gain an understanding of the reach and popularity of the different tools and resources. A survey on the website will allow visitors to provide feedback on the effectiveness of the tools and resources. In addition, a survey of policy and practice stakeholders will be conducted on the usefulness and effectiveness of the tools and resources and on how they have been used and how they have influenced policy and practice.

### Ethics approval

The Community Research Ethics Board (APP7/18/14) has approved this study protocol, and the Research Ethics Board at Ryerson University has deemed that “Based on the information provided the project falls within Article 2.5 of the federal guidelines governing research ethics and so does not require Research Ethics Board review” (REB 2014-263). Participation agreements were signed between the participating project partner agencies. Letters of information will be given out, and written consent will be received by all those engaged in the UV dosimetry study, the interviews, focus groups, and surveys. The letter of information highlights the objective of the study, the composition of the research team, who is eligible to participate, the benefits of being involved in the study, that participation is voluntary, and that all the data collected will remain confidential and accessible only to the research team. It also states that the team expects to finish this study in 2 years’ time (fall of 2016), that we will summarize the study findings and share them with the participating workplaces, and that the results will be put on the project website and the website of the Occupational Cancer Research Centre: http://www.occupationalcancer.ca. We also make the commitment to share our results on posters, at presentations, in newsletters, and in research publications.

### Trial status

Workplaces in the four provinces have been recruited, and a project coordinator, the Sun Safety Advisors, and a project knowledge broker have all been hired. The suite of resources has been completed, and the initial training for the advisors has been completed. The branding of Sun Safety at Work and the creation of the website is underway. Representatives from the partner agencies from the four provinces have met three times in person and continue to have monthly teleconferences. Subgroups have been working on the suite of resources, the hiring process, the creation of the training sessions, and the recruitment of workplaces.

## Summary

Skin cancer represents a significant public health issue for Canada, with heat-stress recognized as an important emerging issue. For both conditions, occupational sun exposure is a significant risk factor. Sun Safety at Work Canada is a unique study in that it aims to develop a nationally applicable, effective, and sustainable sun safety program for outdoor workers that will address both skin cancer and heat illness prevention and can be implemented by individual workplaces and embedded into their existing occupational health and safety systems. This uncontrolled before-after cross-case comparison design does have its limitations in comparison to more rigorous designs, but it is a reasonable response to the realities of real-world research in workplaces where there is no social, legal, or economic imperative to adopt best practices or incorporate evidence-based research. Our expectations remain that the evaluation will provide a detailed understanding of the process of implementing workplace health interventions of evidence-based best practices. The dissemination of the findings will be guided by a comprehensive knowledge translation strategy that will allow for wide distribution of the project’s learnings as a way of influencing policy and practice. Part of this knowledge translation strategy is the use of conceptual frameworks of knowledge transfer to guide workplace interventions and research activities. This model of research, policy, and practice integration, in addition to the theoretically grounded, evidence-based, consultative approach to implementation science is respectfully offered to the research community as a model for workplace intervention research.
